# NF-κB Signaling in Prostate Cancer Progression: Inflammatory Mediators, Survival Pathways, and Regulatory Axes

**DOI:** 10.3390/cancers18081227

**Published:** 2026-04-13

**Authors:** Ranyah Al-Hakm, Alaa Muayad Altaie, Anania Boghossian, Riyad Bendardaf, Iman M. Talaat, Rifat Hamoudi

**Affiliations:** 1 Research Institute of Medical and Health Sciences, University of Sharjah, Sharjah P.O. Box 27272, United Arab Emirates; u22104866@sharjah.ac.ae (R.A.-H.); alaa.abed@sharjah.ac.ae (A.M.A.); aboghossian@sharjah.ac.ae (A.B.); riyad.bendardf@uhs.ae (R.B.); 2 Clinical Sciences Department, College of Medicine, University of Sharjah, Sharjah P.O. Box 27272, United Arab Emirates; 3 Center of Excellence for Precision Medicine, Research Institute of Medical and Health Sciences, University of Sharjah, Sharjah P.O. Box 27272, United Arab Emirates; 4 Oncology Unit, University Hospital Sharjah, Sharjah P.O. Box 72772, United Arab Emirates; 5 Center of Excellence for Cancer Research, Research Institute of Medical and Health Sciences, University of Sharjah, Sharjah P.O. Box 27272, United Arab Emirates; 6 Pathology Department, Faculty of Medicine, Alexandria University, Alexandria P.O. Box 21131, Egypt; 7 Biomedically Informed Artificial Intelligence Laboratory (BIMAI-Lab), University of Sharjah, Sharjah P.O. Box 27272, United Arab Emirates; 8 ASPIRE Precision Medicine Research Institute Abu Dhabi, University of Sharjah, Sharjah P.O. Box 27272, United Arab Emirates; 9 Division of Surgery and Interventional Science, University College London, London NW3 2PS, UK

**Keywords:** prostate cancer, NF-κB signaling, apoptosis resistance, BCL-2, BCL-3, castration-resistant prostate cancer

## Abstract

Prostate cancer (PC) progression is increasingly recognized as a dynamic, inflammation-driven process in which chronic immune dysregulation contributes to disease aggressiveness and therapeutic resistance. Among inflammatory signaling pathways, nuclear factor kappa B (NF-κB) has been consistently implicated in prostate tumorigenesis, castration resistance, and apoptosis resistance. However, despite the extensive literature on NF-κB biology, the mechanisms by which its dysregulation is sustained and functionally shaped during PC progression remain incompletely understood. This review synthesizes current evidence on prostate cancer-specific NF-κB signaling, with an emphasis on stage-dependent activation, molecular regulation within the tumor microenvironment, and downstream transcriptional programs linked to survival and treatment resistance. Particular attention is given to the regulatory role of B-cell lymphoma-3 (BCL-3), an atypical nuclear IκB protein, and its potential interplay with B-cell lymphoma-2 (BCL-2), a well-established NF-κB-regulated anti-apoptotic factor in PC. Available clinical, molecular, and transcriptomic data support constitutive NF-κB activation across multiple stages of PC, particularly in advanced and castration-resistant disease. Although BCL-2 overexpression is well documented as a mediator of apoptosis resistance in PC, evidence directly linking BCL-3 to BCL-2 regulation in this disease remains limited. Data from other malignancies suggest that BCL-3 can modulate NF-κB transcriptional output and enhance BCL-2 expression; however, prostate-specific mechanistic validation is lacking. We propose a testable, hypothesis-driven model in which BCL-3 may function as a context-dependent regulator of NF-κB-mediated survival signaling in PC, potentially influencing BCL-2 expression and therapeutic resistance. However, this relationship remains speculative and is not yet supported by direct mechanistic evidence in PC. By distinguishing between established evidence and inferred mechanisms, this review highlights critical knowledge gaps and outlines experimental strategies to clarify the functional relevance of the BCL-3/BCL-2 axis. Improved understanding of NF-κB regulatory dynamics may inform the development of more precise, stage-adapted therapeutic strategies for advanced PC.

## Simple Summary

Prostate cancer progression is increasingly recognized as a process influenced by chronic inflammation within the tumor environment. Among the key regulators of inflammatory signaling is nuclear factor kappa B, a pathway known to promote tumor growth, survival of cancer cells, and resistance to treatment. Although this pathway has been widely studied in prostate cancer, the mechanisms that sustain its activity during disease progression are not fully understood. This review examines current knowledge on inflammatory signaling in prostate cancer, with particular focus on the regulatory roles of the proteins BCL-3 and BCL-2. BCL-2 is known to help cancer cells avoid cell death, while the contribution of BCL-3 to this process in PC remains less clearly defined and may involve indirect regulatory mechanisms that require further investigation. By summarizing available clinical and molecular evidence, this work highlights important gaps in knowledge and proposes potential mechanisms linking these pathways. A better understanding of these interactions may support future research and the development of improved therapeutic strategies for advanced prostate cancer.

## 1. Introduction

Prostate cancer (PC) remains a major health concern worldwide, affecting millions of men each year. It is the second most frequently diagnosed cancer and the fifth leading cause of cancer-related deaths among men globally, accounting for more than 1.4 million cases and 396,000 deaths in 2022 [1,2]. A hallmark of PC is its pronounced clinical and biological heterogeneity, reflected in significant variability in incidence, age of onset, disease aggressiveness, therapeutic response and mortality across populations [3]. Clinically, PC is frequently characterized by an indolent clinical course: often the time from initial diagnosis to death ranges from 15 to 17 years, particularly in low- to intermediate-grade tumors, resulting in a lower mortality rate despite its high incidence [4]. While this prolonged latency period enables active surveillance and early treatment planning, it also makes it challenging to identify early biomarkers that can reliably distinguish indolent tumors from aggressive or lethal forms of the disease.

Although it’s often a slow progression disease, a subset of PC evolves toward high-grade, locally advanced, metastatic, and ultimately castration-resistant states [5]. This progression is driven by the effect of genetic and epigenetic alterations, tumor microenvironment (TME) remodeling, and aberrant activation of oncogenic signaling pathways, which collectively enable PC cells to bypass androgen dependence and develop resistance to conventional therapies [6]. Defining the molecular drivers of this transition is critical to improve high-grade tumor detection and optimize treatment strategies.

PC treatment strategies are determined based on Prostate-Specific Antigen (PSA) levels, tumor stage, and Gleason grading. Current management includes active surveillance for low-risk diseases to monitor progression and guide therapeutic decision-making, as well as hormonal therapies, immunotherapy, targeted therapy, and chemotherapy for advanced diseases [5,6]. However, disease progression and treatment resistance remain major clinical challenges, highlighting the need to explore therapeutic strategies targeting key signaling pathways involved in PC progression.

Increasing evidence implicated chronic inflammation and immune dysregulation as key drivers of PC initiation and progression. Epidemiological, histological, and molecular studies have linked sustained prostatic inflammation with increased PC risk [7,8]. Chronic inflammatory state promotes cell growth, blood vessel formation, genomic instability and cell death evasion, all of which possess potential implications for disease progression [7]. However, prostate inflammation is often chronic and asymptomatic, especially in the early stages [9], making it challenging to pinpoint the exact causes of inflammation and immune system imbalance. Finding precise biomarkers correlated with PC initiation and progression is critical for advancing disease understanding and developing effective targeted therapies.

During PC progression, inflammatory signaling interacts with key survival pathways, thereby supporting tumor cell survival under stress, immune evasion, and therapeutic resistance. Among the key inflammatory pathways involved in PC is the nuclear factor kappa B (NF-κB) signaling pathway, which regulates the expression of genes involved in immune responses, cell survival, and oncogenesis [10]. Dysregulated NF-κB activity has been consistently observed in prostate tumor tissues and is associated with disease progression, altered androgen signaling, and resistance to conventional therapies [11,12,13,14,15]. Persistent NF-κB activation in PC reflects aberrant regulatory control rather than a transient response to inflammatory signals.

Despite extensive investigations, critical questions remain unanswered, including which pathway components sustain chronic activation in PC, how NF-κB signaling dynamics vary across disease stages, and whether NF-κB activation alone is sufficient to induce metastatic transformation. Additionally, the slow and heterogeneous nature of PC further poses a major challenge for identifying NF-κB-associated biomarkers that reflect chronic rather than acute inflammatory signaling [16]. Extensive research concentrated on canonical NF-κB signaling components. Comparatively limited attention was given to the role of regulatory modulators in controlling transcriptional specificity and downstream effects in PC. Within this regulatory landscape, BCL-3 functions as a context-dependent regulator of NF-κB signaling, where it can modulate transcriptional programs associated with cell survival, including the expression of key anti-apoptotic genes such as BCL-2 [17]. The precise regulatory role and interactions of these molecules in PC remain incompletely defined.

To address these gaps, this review focuses on the role of NF-κB signaling and the complex interactions among its molecular components in PC development, emphasizing how BCL-3 and BCL-2 interact and contribute to survival and therapeutic resistance. This focus is particularly relevant, given that PC is a slow-growing disease that can progress silently over years to aggressive metastatic forms, where its pathogenesis is believed to be strongly influenced by chronic inflammatory microenvironments and a dysregulated immune system. To ensure a focused and comprehensive synthesis of relevant evidence, a structured literature search strategy was employed, as outlined below.

## 2. Literature Search Strategy

This narrative review was informed by a structured literature search of PubMed, Scopus, and Web of Science. The search covered studies published between January 2010 and March 2024 and used combinations of the following keywords: “prostate cancer,” “NF-κB,” “nuclear factor kappa B,” “BCL-3,” “BCL-2,” “inflammation,” “apoptosis,” “castration-resistant prostate cancer,” and “therapeutic resistance.” Studies were prioritized if they provided prostate cancer-specific evidence, including analyses of human prostate tissues, patient-derived datasets, prostate cancer cell lines, xenograft models, or clinically relevant disease states such as castration-resistant prostate cancer. Foundational mechanistic studies from other cancer types were included only when they offered biologically relevant insights into NF-κB or BCL-3/BCL-2 regulation that may inform PC biology. The reference lists of key articles were manually screened to ensure comprehensive coverage of the relevant literature.

## 3. Prostate Cancer: Epidemiology, Clinical Features, and Molecular Basis

### 3.1. Epidemiology of Prostate Cancer

The global burden of PC is expected to increase in the coming decades due to population aging and demographic changes such as economic growth, with estimates reaching 2.88 million cases and 940,000 deaths by 2050 [18]. Despite its high incidence, out of 10 million men diagnosed with PC worldwide, only 700,000 progress to the metastatic stage [19]. However, recent epidemiological trends are concerning, with a documented rise in metastatic PC incidence, including an annual increase of 5.3% between 2010 and 2018 in the United States [20]. This shift has significant clinical implications, as the 5-year relative survival rate declines sharply from 97.1% for localized disease to approximately 30% in metastatic cases [21], indicating that the increase in metastatic cases is likely to translate into increased PC-related deaths over time if not effectively addressed.

### 3.2. Early-Onset Prostate Cancer and Diagnostic Challenges

Marked variability in PC incidence and outcomes reflects the interplay of multiple risk factors, including age, ethnicity, genetic susceptibility, and environmental exposures [8,22,23,24]. Although PC has typically been considered a disease of older men, recent reports indicate an increasing incidence among younger populations, including men under 55 and, increasingly, under 40 years of age [25,26,27,28]. Notably, younger patients are often diagnosed with more aggressive and advanced disease, suggesting distinct biological mechanisms compared to late-onset PC [28,29]. This trend could be due to a combination of factors, including underdiagnosis, racial and genetic influences, a higher prevalence of human papillomavirus (HPV) infection among younger men, obesity, and exposure to environmental carcinogens [28]. Despite the availability of various diagnostic and prognostic biomarkers, including PSA, considerable uncertainty remains regarding their sensitivity and specificity. This is particularly evident when distinguishing benign prostatic conditions from early-stage or malignant lesions, due to the similarity between the effects of gland injury and PC growth [30]. PSA screening, although widely used, is associated with high false-positive rates, leading to overdiagnosis and overtreatment [31]. These limitations highlight the need for more accurate biomarkers that reflect the molecular mechanisms driving PC initiation and progression.

### 3.3. Occurrence and Progression of Prostate Cancer

PC is a non-cutaneous malignant neoplasm, its development driven by dynamic interactions between molecular pathways and the tumor microenvironment (TME) [3]. Prostate tumor growth is initially androgen-dependent, relying on androgen receptor (AR) signaling for proliferation and survival [32]. However, as the disease advances, tumor cells may transition from androgen-dependent to androgen-independent prostate cancer (AIPC) [33]. Mechanisms underlying this progression include AR amplification and overexpression, AR mutation, the expression of AR splice variants such as AR-V7, and intracrine androgen biosynthesis [34,35]. Clinically, the progression of PC is reflected by increasing histopathological grade and tumor stage, commonly assessed using the Gleason grading system, TNM classification, and PSA levels [3,36,37]. These molecular and biological alterations underpin the histopathological grading and clinical staging systems used to evaluate prostate cancer progression, as described in the following section.

### 3.4. Prostate Cancer Classification: Grading and Staging

**Figure 1:**
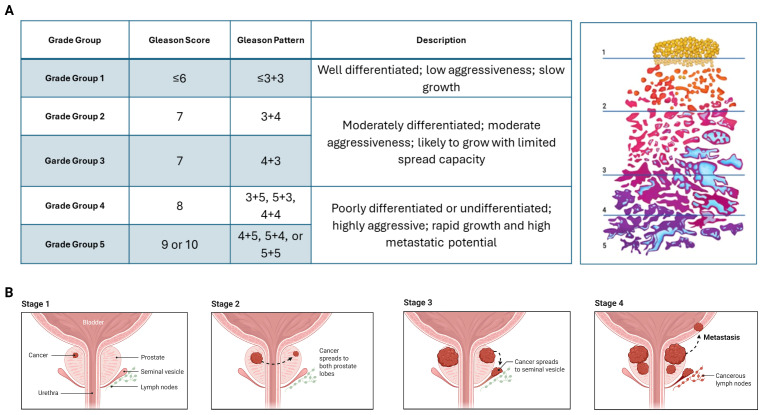
Grading and staging prostate cancer. (**A**) The ISUP Gleason Grade Group system. (**B**) Clinical tumor staging system. Created in BioRender. Alhakm, R. (2026) https://BioRender.com/oarqfe2 (accessed on 12 March 2026).

PC grading follows the Gleason scoring system provided by the International Society of Urological Pathology (ISUP) [38,39]. According to ISUP, PC is classified into five grade groups ranging from well-differentiated (grade 1) to poorly differentiated tumors (grade 5). The Gleason system evaluates two predominant histological patterns; each scored from 1 to 5 based on cellular differentiation. The sum of these patterns yields the Gleason score, (6–10), reflecting tumor aggressiveness [40]. A summary of the grade group system is provided in Figure 1A. In addition to grading, PC is classified into four clinical stages. As shown in Figure 1B, prostate cancer is classified into four stages: stage 1 and 2 represent organ-confined disease of increasing tumor size, stage 3 indicates locally advanced tumors extending beyond the prostate, and stage 4 corresponds to metastatic disease involving distant sites such as lymph nodes, lung, liver, and bone [39,41,42].

## 4. Inflammatory Contexts of Prostate Cancer

While PC is often characterized by slow tumor growth, where the development of a 0.4-inch tumor typically occurs over a period of 4 to 10 years, chronic inflammation within the prostate plays a central role in initiating neoplastic transformation and promoting disease progression [3].

### 4.1. Initiation of Prostatic Inflammation

In fact, since 1863, inflammation has been linked to cancer development [43]; epidemiological studies indicate that inflammatory conditions, such as hepatitis driven by hepatitis viruses, Helicobacter pylori-induced gastritis, and inflammatory bowel diseases like colitis, are associated with increased cancer risk and therefore recognized as a hallmark of cancer [44,45,46]. Factors such as urine reflux, hormonal imbalance, infection (e.g., Escherichia coli), and viral agents like HPV have been implicated in inducing prostatic inflammation through sustained immune cell infiltration and cytokine production [9,47,48]. Although infection has been reported as an initiator of inflammation in PC, and the presence of HPV in PC tissues suggests activation of inflammatory pathways and a potential contribution to pathogenesis, its association with PC risk is still debated, where the exact role remains controversial and requires further investigation [48,49,50,51]. Initially, immediate activation of the immune response contains the unusual growth of malignant cells, leading to the indolence of PC in many cases [52]. However, these cells can escape immunosurveillance and develop mechanisms to utilize inflammatory signaling pathways to support their growth [53]. Unresolved chronic inflammatory response contributes to the initiation, progression, immune evasion, and treatment resistance of PC and other diseases; however, the underlying mechanisms are yet to be fully understood [54,55,56,57].

### 4.2. Chronic Inflammation in Prostate Tissue

Chronic inflammation is an aberrant, prolonged immune response resulting from persistent infections or autoimmune reactions. Chronic inflammation drives various cellular and molecular alterations, including fibrosis, genetic variants, immune suppression, aberrant cytokine and chemokine secretion, sustained activation of NF-κB and Signal Transducer and Activator of Transcription 3 (STAT3) pathways, and epigenetic modifications. Although numerous studies have linked chronic inflammation to PC pathogenesis, the exact triggers and mechanisms underlying the initiation and persistence of prostatic inflammation remain unclear [53,58,59].

The pathogenesis process is initiated by cell proliferation, chronic inflammation, and microbial infection, resulting in the generation of reactive oxygen species (ROS) and the infiltration of inflammatory cells, which induce DNA damage [3,9,60]. These events lead to the development of a proliferative inflammatory atrophy state. This state is associated with chronic inflammation via the release of the cyclooxygenase-2 (COX-2) enzyme and pro-inflammatory prostanoids (e.g., prostaglandin E_2_), which mediate oxidative stress [61,62]. Sustained inflammatory signaling induces DNA damage and genomic instability. These alterations promote progression to prostatic intraepithelial neoplasia (PIN), a recognized precancerous state of localized PC. As the PC progresses, the TME becomes infiltrated with immunosuppressive cells such as myeloid-derived suppressor cells (MDSCs) and regulatory T cells (Tregs) that promote immune evasion [62,63]. These changes enable invasion and metastasis of cancerous cells [64,65,66].

Genetic alterations in PC not only promote tumor initiation but also modulate the TME via inflammatory signaling [6]. For example, structural chromosomal rearrangements, such as TMPRSS2-ERG gene fusion, observed in around 40–60% of patients, alter the expression of proinflammatory cytokines and genes. These include genes involved in invasion and metastasis, such as C-X-C motif chemokine receptor 4 (CXCR4), matrix metallopeptidase 9 and 3 (MMP9, MMP3), and ADAM metallopeptidase with thrombospondin type 1 motif 1 (ADAMTS1). Additionally, these rearrangements also influence AR target genes such as PSA, prostate-specific membrane antigen (PSMA, gene symbol FOLH1), and solute carrier family 43 member 1 (SLC43A1) [67,68,69]. Collectively, these changes promote an inflammatory tumor microenvironment that supports cancer progression [70]. PTEN loss activates the PI3K/AKT/mTOR pathway, promoting proliferation, survival, and metabolic reprogramming [71,72,73]. However, a gap remains in our understanding of the mechanistic links between genetic alterations and inflammatory signaling. Elucidating how functionally relevant genetic variants shape the inflammatory landscape at different stages of PC could reveal novel therapeutic targets.

The role of inflammation in cancer is dynamic and context-dependent, acting initially as a protective response but, when sustained, evolving into chronic immune dysregulation. The chronic inflammatory state concomitantly activates various signaling cascades, among which dysregulation of NF-κB constitutes a defining characteristic of prostatic inflammation and a pivotal determinant in the pathogenesis of PC [74,75]. Studies highlight the association between constitutive NF-κB activation, prostatic inflammation, and PC progression via interaction with oncogenic pathways, including AR signaling [75,76,77]. Thus, elucidating the molecular mechanism of the NF-κB pathway will help shed light on the initiation and progression of the disease.

## 5. NF-κB Signaling in Prostate Cancer: A Context-Dependent Framework

The NF-κB pathway is a highly regulated signaling cascade with a multi-layered regulatory structure divided into input, processing, and output layers, ensuring a tightly controlled and transient response to various cellular and environmental stimuli. The layered regulation supports cellular homeostasis under physiological conditions while facilitating adaptive responses to stress, pathogens, or tissue injury. NF-κB signaling comprises two major pathways, canonical and non-canonical, which differ in their upstream activators, molecular mediators, and transcriptional outputs [78].

### 5.1. Canonical and Non-Canonical NF-κB Pathways

**Figure 2:**
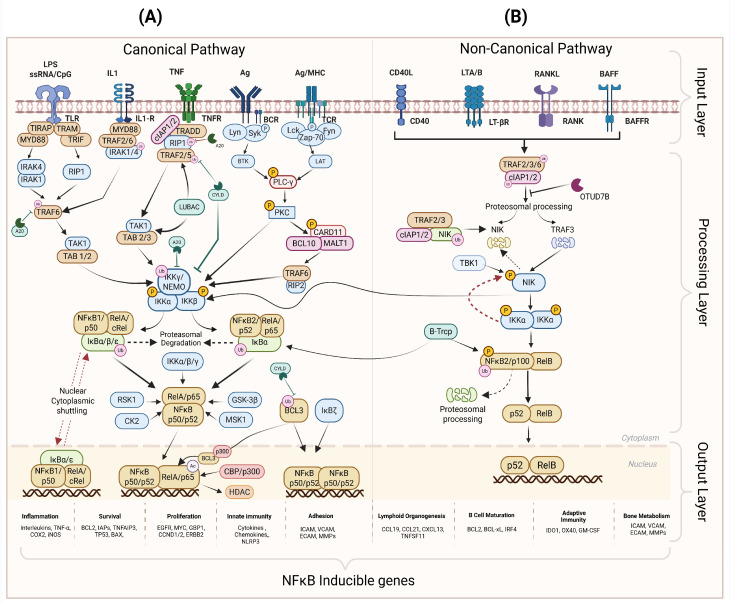
Nuclear factor kappa B (NF-κB) signaling pathway. The NF-κB pathway can be organized into three layers—input, processing, and output—and is activated through the canonical and non-canonical pathways. (**A**) In the canonical pathway, extracellular stimuli trigger upstream adaptor proteins, which in turn activate the IKK complex. This leads to phosphorylation and degradation of IκB inhibitors, releasing NF-κB heterodimers to translocate to the nucleus, regulating the expression of target genes involved in inflammation, cell survival, proliferation, and immunity. (**B**) In the non-canonical pathway, activation by receptors such as BAFFR, CD40, RANK, or LTβR stabilizes NIK, which phosphorylates IKKα homodimers. This triggers proteolytic processing of p100 into p52, allowing RelB/p52 heterodimers to accumulate, translocate to the nucleus, and regulate the expression of genes associated with lymphoid organ development, B-cell maturation, and adaptive immune responses. Created in BioRender. Alhakm, R. (2026) https://BioRender.com/xmmm0zx (accessed on 12 March 2026).

Pathway activation is initiated at the first layer, known as the input layer, where membrane and intracellular receptors, such as cytokine receptors, pattern recognition receptors (PRRs), antigen receptors, and DNA damage sensors, detect various stimuli, including inflammatory cytokines, microbial components, growth factors, and cellular stress. Based on the nature of the activating signal and the cellular context, either the canonical or non-canonical NF-κB pathway is activated; each control distinct but complementary transcriptional and cellular responses [79,80] (Figure 2).

The second layer is the processing layer; upon receptor stimulation, adaptor proteins such as TRADD, MyD88, TRIFs, and TRAFs are recruited to trigger intracellular signaling which activates the IκB kinase (IKK) complex, composed of IKKα, IKKβ, and regulatory subunit IKKγ (NEMO) [81]. In antigen receptor signaling, a specific transient complex known as the CARD11–BCL10–MALT1 complex (CBM complex), mediates and sustains pathway activity via MALT1-dependent cleavage of the negative regulatory protein A20 [82]. In the physiological state, NF-κB/Rel proteins composed of (RelB, c-Rel, RelA (p65), NF-κB1 (p105), and NF-κB2 (p100)), formed various homodimer or heterodimer complexes and were retained in the cytoplasm by inhibitor κB (IκB) family members (IκBα, IκBβ, IκBε, IκBζ, BCL-3, and IκBNS), maintaining transcriptional inactivity [79,81]. The IκB protein family plays a critical role in the feedback regulation of the NF-κB activity, maintaining the physiological state of the cells (Figure 2) [83,84,85]. In the canonical pathway, IKKβ phosphorylates IκBα at specific serine residues (Ser32 and Ser36), triggering k48-linked ubiquitination and proteasomal degradation. This release predominantly RELA (p65/p50) heterodimers, exposing their nuclear localization signals (NLS) and enabling rapid nuclear translocation [81,86]. Interestingly, activation of the non-canonical pathway occurs at a slower rate compared to the canonical pathway. In the non-canonical pathway, upstream kinases like NF-κB-inducing kinase (NIK) mediate the phosphorylation of IKKα complexes, which leads to the processing of p100 into p52, allowing p52/RelB nuclear translocation as shown in Figure 2 [87,88]. While the canonical pathway mainly regulates the immune response, cell proliferation, and cell survival, the non-canonical pathway controls the expression of the genes involved in lymphoid organization and body homeostasis-related genes, as indicated in Figure 2 [79,89].

Lastly, the third layer, also known as the output layer, represents the transcriptional regulation layer. Upon nuclear translocation, NF-κB transcription factors bind to κB enhancer elements within target gene promoters in association with co-activators CBP/p300 or co-repressors Histone Deacetylases (HDACs) and regulate the gene transcription dynamics of more than 400 inducible genes [80,81]. Thus, modulating a switch between various biological processes, including immune response, stress response, inflammation, cell growth, cell survival, B-cell development, and lymphoid organogenesis [78,90]. Importantly, NF-κB signaling is functionally defined by subunit composition rather than pathway activation alone. Notably, canonical RELA/p50 complexes regulate transcription of anti-apoptotic genes, where genetic study demonstrates the loss of RELA and c-Rel results in reduced BCL-2 expression, linking NF-κB activation directly to apoptosis resistance [91]. In contrast, p50 or p52 homodimers lack intrinsic transactivation domains; however, evidence indicates that they can directly enhance BCL-2 transcription through promoter binding in a context-dependent manner [92]. Thus, NF-κB transcriptional activity is determined by subunit composition, post translational modification, the recruitment of specific co-regulators and cellular context. This mechanistic complexity underlies the context-dependent nature of NF-κB signaling and highlights the importance of subunit-specific transcriptional programs in shaping cell survival and disease progression.

### 5.2. NF-κB Regulatory Proteins

The NF-κB pathway is tightly regulated through negative feedback mechanisms to maintain homeostasis, ensure transient signaling, and prevent constitutive activation. Key regulators include A20/TNFAIP3, which mediate the deubiquitination of signaling components to inhibit NF-κB activation [93]; IκBα resynthesis, which transiently sequesters NF-κB/Rel complexes in the cytoplasm [94]; and CYLD-dependent inhibition, which further dampens NF-κB by cleaving ubiquitin-activating modifications [95]. Furthermore, a crucial and key regulator of the NF-κB pathway activity is BCL-3. BCL-3 fine-tunes transcriptional outcomes by acting either as a transcription co-activator or inhibitor. It acts as a transcription co-activator by stabilizing nuclear p50/p52 homodimers and as an inhibitor by retaining NF-κB complexes in the cytoplasm, according to their cellular context and PTMs [96]. Nonetheless, these negative feedback mechanisms can be disrupted through delayed resynthesis of pathway inhibitors, mutations in pathway regulatory genes, or chronic stimulation of upstream receptors/adaptors [97,98,99,100]. These mechanisms further potentiate NF-κB hyperresponsiveness and sustain prolonged activation of the pathway, thereby establishing a pathogenic positive feedback loop that perpetuates oncogenic transcriptional programs.

### 5.3. NF-κB Dysregulation as a Key Mechanism for Cancer Progression

**Figure 3:**
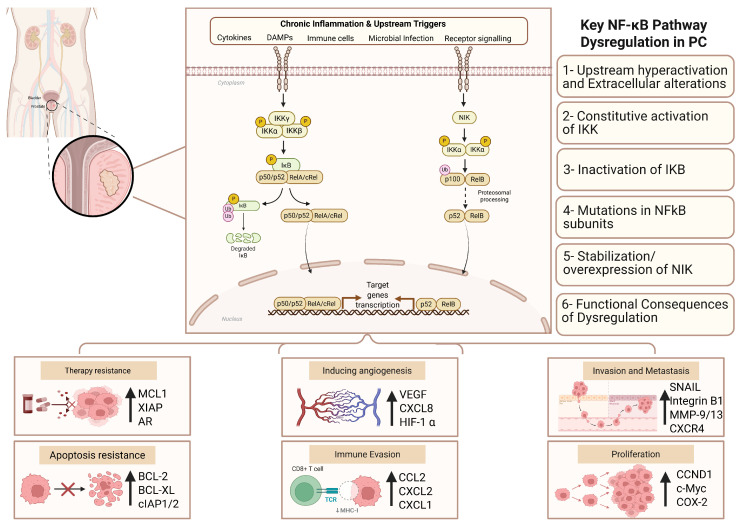
Key dysregulations of the NF-κB pathway in prostate cancer. Aberrant activation of the NF-κB pathway contributes to PC progression through multiple molecular alterations. Upstream hyperactivation and persistent receptor signaling induced by aberrant extracellular stimuli lead to sustained activation of the IKK complex. Dysregulated expression of NF-κB pathway proteins due to mutations or constitutive activation of IKK promotes continuous degradation of IκB, resulting in persistent nuclear translocation of NF-κB dimers. Also, mutations causing IκB inactivation, similarly, result in continuous free NF-κB dimers that translocate and accumulate in the nucleus. Additionally, mutations in NF-κB subunits enhance DNA binding and sustain oncogenic NF-κB transcriptional activity. All of these alterations drive disease progression by enhancing tumor proliferation (CCND1, MYC, COX-2), metastasis and invasion (SNAIL, ITGB1, MMP-9/13, CXCR4), resistance to apoptosis (BCL-2, BCL-XL, BIRC2, BIRC3), therapy resistance (MCL1, XIAP, AR), inducing angiogenesis (VEGF, CXCL8, HIF1A), and immune evasion (CCL2, CXCL2, CXCL1). Created in BioRender. Alhakm, R. (2026) https://BioRender.com/uhz01ie (accessed on 12 March 2026).

The NF-κB pathway is tightly regulated under physiological conditions, where transient activation supports cellular homeostasis and immune responses; however, dysregulation leads to sustained activation, shifting NF-κB signaling toward pathological outcomes associated with inflammatory and neoplastic diseases [10,101]. Under sustained activation, NF-κB exhibits pro-tumorigenic activity arising from genetic alterations—such as amplifications of NF-κB subunits and genetic variants in IκB inhibitors—as well as from chronic exposure to inflammatory environments driven by the secretion of inflammatory cytokines and ROS [102]. In chronic inflammatory conditions, sustained NF-κB signaling establishes positive feedback loops that sustains constitutive NF-κB signaling and fosters an immunosuppressive microenvironment, contributing to the pathological state [80,103]. Mechanistically, within the tumor microenvironment, sustained NF-κB activation promotes tumorigenesis by remodeling cellular metabolism through the hexokinase 2-mediated Warburg effect, promoting angiogenesis through HIF-1α/VEGF signaling, evading apoptosis by upregulating BCL-2, and accelerating cell cycle progression through cyclin D1 induction [80,90]. These processes have supported the well-established role of NF-κB dysregulation in disease development, treatment resistance, and progression to aggressive stages since its early discovery (Figure 3) [101,102]. However, these effects are highly context-dependent, as NF-κB signaling may also contribute to normal cellular survival and immune regulation under physiological conditions.

Numerous studies have confirmed that NF-κB dysregulation influences cancer hallmarks and is implicated in various types of cancers, including breast, colorectal, and PC [104,105,106,107,108]. Despite extensive research, the temporal and spatial regulation of NF-κB activity within the tumor microenvironment and its influence on tumor initiation, progression, and therapeutic response remain poorly understood, particularly given its context-dependent roles that vary across disease stages, cellular contexts, and microenvironmental conditions, ranging from protective to tumor-promoting functions. The subsequent sections, therefore, examine NF-κB dysregulation in PC, focusing on its activation mechanisms, effects on cellular processes, and contributions to tumor progression and therapy resistance.

### 5.4. Receptor-Driven Inflammatory Inputs Sustaining NF-κB Activation in Prostate Cancer

Chronic stimulation of upstream signaling receptors in the input layer sustains NF-κB activation in PC. Pattern-recognition receptors and inflammatory cytokine receptors—including Toll-like receptors (TLRs), tumor necrosis factor receptor (TNFR), and interleukin-1 receptor (IL-1R)—act as key nodes that integrate inflammatory signals from the TME, therapy promoting disease development and progression. Among these, TLRs are frequently dysregulated in PC and contribute to persistent NF-κB signaling. Elevated TLR4 expression in PC tissues has been associated with increased tumor proliferation, invasion, and a higher Gleason grade [109] mechanistically through MyD88-dependent NF-κB activation and sustained production of pro-inflammatory cytokines such as interleukin-6 (IL-6) and IL-1β and genes involved in tumor progression [110,111]. Similarly, TLR9 promotes immune evasion via recruitment of MDSCs and activation of STAT3, while also enhancing angiogenesis and invasion through upregulation of VEGF, MMPs, and COX-2 [112,113,114,115]. TLR3 activation has also been associated with enhanced chemoresistance, migration, and invasion through upregulation of IL-6, IL-8, and IFN-β [116]. Notably, increased expressions of TLR3, TLR4, and TLR9 correlate with disease recurrence, highlighting their role as upstream modulators of NF-κB signaling [117] (Figure 3).

TNFR signaling represents another major inflammatory input shaping NF-κB activity in PC. TNF-α exerts context-dependent effects and displays both tumor-promoting and inhibitory roles, depending on its concentration and environmental context [118]. Chronic low-level TNF-α exposure predominates within the TME and promotes tumor progression by enhancing NF-κB-dependent transcription of genes associated with invasion and metastasis (MMP-9, Snail, and fibronectin), and angiogenesis (IL-6, VEGF, and IL-8) [108,119,120,121], while also stimulating the development of castration resistance [122]. Conversely, high-level TNF-α signaling can induce apoptosis and anti-tumor immunity [123,124,125]; however, in the chronic inflammatory environment of PC, tumor-promoting effects predominate.

IL-1R-mediated NF-κB signaling via IL-1α/β further illustrates the context-dependent role of inflammatory inputs in PC. While IL-1α induces cell cycle arrest [126], and IL-1β activates Th1 and Th17 anti-tumor immunity [127], their pro-tumorigenic roles dominate in PC by enhancing angiogenesis, invasiveness, castration-resistant prostate cancer (CRPC) progression, and metastasis [128,129,130,131] (Figure 3).

Beyond innate immune receptors, adaptive immune signaling pathways, particularly BCR and TCR, may indirectly contribute to NF-κB activation in PC, although mechanistic evidence remains limited. Chronic activation of BCR/TCR within the TME sustains pro-inflammatory cytokine production, therapy amplifying NF-κB-dependent survival and proliferative programs [132]. Furthermore, lymphotoxin (LT) induces stromal fibroblasts to secrete C-X-C Motif Chemokine Ligand 13 (CXCL13), facilitating the recruitment of immunosuppressive myeloid cells into the TME and further amplifying NF-κB signaling within the PC [133]. Collectively, these receptor-mediated inflammatory inputs establish a sustained NF-κB signaling environment that supports tumor survival and progression. These findings highlight that receptor-mediated NF-κB activation is highly context-dependent, with outcomes shaped by ligand intensity, duration of signaling, and the tumor microenvironment.

### 5.5. Stage-Dependent NF-κB Activation in Prostate Cancer

NF-κB activation in PC is highly dynamic and varies according to disease stages, histological grade, and AR signaling status. Both canonical and non-canonical NF-κB pathways have been shown to contribute to disease progression, therapy resistance, and the transition from localized PC to CRPC. In localized PC, NF-κB activation is predominantly driven by chronic inflammatory signaling within the TME. Increased nuclear NF-κB/p65 staining and elevated IκBα expression are consistently observed in PC tissues and correlate with higher tumor grade [134]. The transgenic mouse model further demonstrates a progressive increase in p65 and p50 expression during disease progression [135]. This activation is reinforced by inflammatory pathways such as IL-6/ERK signaling, which show a stepwise increase in NF-κB activity from benign conditions to malignant disease [136].

During progression to androgen-independent states, disruption of AR signaling leads to compensatory NF-κB activation. Androgen-independent PC cell lines (PC-3 and DU-145) exhibit higher constitutive NF-κB activity compared with androgen-dependent LNCaP cells and normal prostate epithelial cells, highlighting the association between NF-κB activation and castration resistance [137]. In CRPC, canonical NF-κB signaling driven by IKKβ promotes tumor survival by enhancing stemness (e.g., Nanog, Oct4, Sox2), epithelial-to-mesenchymal transition (EMT), and anti-apoptotic pathways via survivin and Ki67 [138].

Non-canonical NF-κB signaling plays a critical role in advanced and metastatic PC. Activation of the LTβR–RelB/p52 axis by tumor-infiltrating immune cells promotes metastasis and castration resistance [139]. In parallel, nuclear IKKα and STAT3 signaling further promotes metastasis by repressing the tumor suppressor Maspin and upregulating MMPs [133,139,140]. Mechanistically, RelB enhances invasion and metastasis by upregulating the expression of Integrin B-1, which promotes extracellular matrix adhesion [141]. RelB activity is also strongly associated with higher Gleason scores and aggressive tumor phenotypes, while inhibition in androgen-independent DU145 cells reduces IL-8 secretion and suppresses tumor growth [11,142]. To further highlight its role in CRPC, inhibition of RelB/p52 nuclear activity has been shown to reduce tumor growth and increase radiosensitivity [143,144,145]. These findings highlight the therapeutic relevance of targeting non-canonical NF-κB signaling in advanced PC.

NF-κB also contributes to CRPC through direct crosstalk with AR signaling. In androgen-deprivation-resistant xenograft models, NF-κB has been shown to contribute to persistent AR signaling, sustaining PC cell proliferation and survival despite androgen-depleted conditions [146]. NFκB was found to also promote CRPC progression by inducing constitutively active AR splice variants (ARVs), which maintain AR signaling and drive tumor growth [147]. Overexpression of p52 (NF-κB2) further enhances AR activation and nuclear translocation via the recruitment of coactivators such as p300, driving the expression of AR-dependent genes and promoting the development of CRPC [148]. Despite its prominent tumor-promoting role in advanced disease, NF-κB activity may exert distinct effects depending on disease stage and cellular context, highlighting its context-dependent function in PC progression.

### 5.6. Transcriptomic Insights

**Figure 4:**
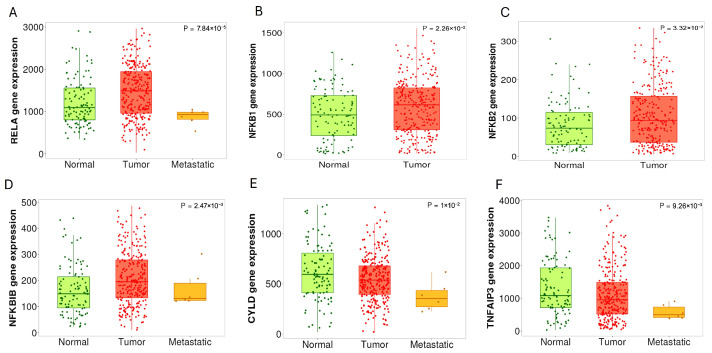
Expression of RELA, NF-κB subunits, NFKBIB, and NF-κB negative regulators in the prostate. Box plots show gene expression in normal prostate tissues (*n* = 106), primary tumors (*n* = 283), and metastatic samples (*n* = 6) from the TNMplot database. Boxes represent the interquartile range (IQR), the center line indicates the median, and whiskers show the minimum and maximum values. Statistical significance was assessed using a two-tailed Mann–Whitney U test with Benjamini–Hochberg correction. (**A**) RELA expression: RELA expression is significantly higher in primary tumors compared to normal tissues (*p* = 1.03 × 10^−4^), suggesting a role in NF-κB signaling activation during prostate cancer progression. A highly significant difference among groups is indicated by *p* = 7.84 × 10^−5^. (**B**,**C**) NF-κB subunits NFκB1 and NFκB2: both NFκB1 and NFκB2 expression are significantly higher in tumor tissues compared to normal tissues (*p* = 2.26 × 10^−2^ and *p* = 3.32 × 10^−2^, respectively), supporting the involvement of the NF-κB pathway in prostate cancer progression. (**D**) NFKBIB (IκBβ) expression: NFKBIB is significantly upregulated in primary tumors compared to normal tissues (*p* = 3.42 × 10^−4^). A highly significant difference among groups is indicated by *p* = 2.47 × 10^−3^, suggesting a potential role in prostate cancer progression. (**E**,**F**) NF-κB negative regulators CYLD and TNFAIP3: CYLD expression decreases progressively in primary and metastatic tissues compared to normal tissues (*p* = 0.01), while TNFAIP3 is downregulated in primary and metastatic tissues (*p* = 0.0093). The downregulation of these negative regulators suggests a mechanism for constitutive NF-κB pathway activation during prostate cancer progression.

Given the established significant role of specific NF-κB subunits and regulators in oncogenesis, we aimed to further investigate and identify the most prominently dysregulated components throughout disease progression by profiling their RNA expression pattern in clinical PC samples. Using the TNMplot tool (Tumor, Normal, and Metastatic), which integrates transcriptomic data from resources including TCGA, GTEx, and GEO, we analyzed differential gene expression within the NF-κB pathway across 106 normal, 283 tumor, and 6 metastatic PC tissues [149]. The data from the TNMplot tool demonstrated that RelA expression is significantly elevated in tumor tissues compared to normal and metastatic tissues (*p* = 7.84 × 10^−5^) (Figure 4A). These findings support the role of RelA in tumorigenesis; the lower expression in metastatic samples may reflect limited sample size (*n* = 6 samples) or a context-dependent shift toward alternative NF-κB subunits.

Additionally, consistent with evidence that dysregulation of NF-κB transcription factors contributes to tumorigenesis, analysis of NF-κB1 and NF-κB2 expression revealed a significant increase in tumor tissues compared to normal tissues (*p* = 2.26 × 10^−2^ and *p*= 3.32 × 10^−2^, respectively) (Figure 4B,C). Suggesting potential activation of both canonical and non-canonical NF-κB pathways. Furthermore, metastatic PC cases show increased nuclear NF-κB staining compared to non-metastatic cases [12]. Although IκBβ (NFKBIB) is an inhibitor of NF-κB signaling, TNMplot data showed that it is upregulated in tumor tissues compared to normal tissues (*p* = 2.47 × 10^−3^) (Figure 4D). This may reflect a compensatory or regulatory feedback response to NF-κB activation, highlighting the complexity of pathway regulation. These findings highlight how dysregulation of the NF-κB subunit contributes to the progression of PC, metastasis, and therapy resistance.

Dysregulated ubiquitination pathways also contribute to NF-κB hyperactivity; for example, reduced expression of CYLD is observed in PC, leading to sustained NF-κB signaling [150]. Furthermore, PSMA7 overexpression or mutation enhances AR transactivation and enhances IκB degradation, thereby amplifying NF-κB activity and contributing to castration-resistant progression [151]. Upon TNMplot analysis, a progressive decrease in CYLD expression was observed in PC tissues, with the highest expression observed in normal tissues (*p* = 1 × 10^−2^) (Figure 4E). Similarly, TNFAIP3 (A20), a negative regulator of the NF-κB pathway, showed decreased expression from normal to metastatic prostate tissues (*p* = 9.26 × 10^−3^) (Figure 4F). The loss of CYLD and TNFAIP3 in PC tissues enhances NF-κB pathway activation, which thereby contributes to disease progression.

While TNMPlot analysis provided preliminary insights into gene expression differences across disease states, the limited representation of metastatic samples reduces the statistical robustness of these observations. Given the marked heterogeneity of metastatic PC, these findings should be considered hypothesis-generating rather than definitive, and require validation in larger, well-characterized cohorts.

### 5.7. Survival Pathways Downstream of NF-κB in Prostate Cancer

NF-κB signaling exerts context-dependent effects, where transient activation supports normal cellular functions, while sustained activation within the tumor microenvironment promotes tumorigenesis. The output layer of NF-κB signaling regulates the transcription of genes that collectively drive tumorigenesis, metastasis, and therapy resistance in PC (Figure 3). NF-κB regulates cell cycle progression and proliferation. At the molecular level, NF-κB regulates key genes involved in cell survival and proliferation, including anti-apoptotic proteins (e.g., BCL-2, BCL-XL, IAPs), cell cycle regulators (e.g., Cyclin D family), and oncogenic drivers such as c-MYC [152,153,154]. In parallel, NF-κB induces pro-inflammatory cytokines, including IL-6, IL-8, and TNF-α, establishing autocrine and paracrine feedback loops that sustain chronic inflammation and reinforce NF-κB activation while concurrently stimulating additional oncogenic pathways [108]. For example, IL-6 activates the JAK/STAT3 and MAPK pathways, which further amplify NF-κB signaling and promote tumor cell survival and therapeutic resistance [155,156]. Experimental studies demonstrate that the chromatin modulator nuclear receptor-binding SET domain protein 2 (NSD2) enhances the expression of NF-κB target genes (e.g., IL-6, IL-8, and TNF-α), and auto-regulates its own expression, creating a positive feedback loop that sustains NF-κB activation and chronic inflammation within the TME [157].

Beyond tumor-intrinsic effects, NF-κB reshapes the TME to support immune evasion and metastasis. It induces chemokines such as CXCL1, CXCL2, and MCP-1, promoting recruitment of immunosuppressive cells including MDSCs and tumor-associated macrophages (TAMs) [158,159]. NF-κB also enhances expression of adhesion molecules (e.g., ICAMs, VCAM-1), facilitating tumor invasion, while IL-8–mediated signaling promotes angiogenesis through MMP-9 activation and myeloid cell recruitment especially in CRPC [160,161]. Additionally, oncogenic alterations such as PKCε and PTEN loss further activate NF-κB signaling, leading to increased COX-2 expression and prostaglandin E2 (PGE2) production, thereby reinforcing inflammation, immune modulation, and disease progression [162]. Overall, dysregulation of NF-κB in PC involves complex alterations across all three layers of the signaling cascade. Targeting key components within the NF-κB pathway offers therapeutic potential to mitigate carcinogenic effects and improve clinical outcomes.

## 6. BCL-3 as an Emerging Regulator of NF-κB Transcriptional Output

While dysregulation of the NF-κB pathway is a well-established driver of PC progression, increasing evidence suggests that subunit-specific interactions within the NF-κB network modulated by regulatory proteins shape downstream signaling outputs; thus, a detailed understanding of the functional interplay of these subunits is crucial. Among these, BCL-3 is a key regulatory component of the NF-κB pathway, which influences the expression of target genes involved in cancer cell survival, including the anti-apoptotic protein BCL-2, which is one of the NF-κB inducible genes that has been implicated in PC progression and therapy resistance [163,164].

### 6.1. Impact of BCL-3 Dysregulation in Prostate Cancer Progression

BCL-3 dysregulation has been implicated in cancer progression by promoting proliferation, invasion, metastasis, and treatment resistance through its direct and indirect regulatory roles in multiple signaling pathways, including the NF-κB pathway [96,165,166]. Altered BCL-3 expression, often arising from aberrant NF-κB activation, is a common feature across multiple solid tumors, including breast cancer [104], ovarian cancer [167], colorectal cancer [168], nasopharyngeal cancer [169], endometrial cancer [170], cervical carcinoma [171], and PC [172]. In PC, BCL-3 has been associated with the recruitment of inflammatory cells such as leukocytes and the enhancement of anti-apoptotic processes, collectively promoting disease progression. A functional study has demonstrated that BCL-3 knockdown reduces PC cell growth and sensitizes cells to apoptosis following chemotherapeutic treatment, supporting its role in therapy resistance, primarily through recruitment of BCL-3 to NF-κB binding sites [172]. Beyond these findings, there is a notable lack of studies investigating BCL-3 in PC, and its precise molecular mechanisms remain poorly understood, highlighting the need for further investigations.

Both proteins could be potential targets for PC, but it remains unclear how they influence the onset and progression of the disease. Although direct evidence of crosstalk between BCL-3 and BCL-2 in PC is lacking, limited evidence from other contexts suggests that BCL-3 may influence BCL-2 expression. Given the central role of NF-κB dysregulation in PC progression, the following sections will focus on the roles and regulation of BCL-3 and BCL-2 and consider the potential for functional interplay in this context.

### 6.2. Context-Dependent Regulatory Functions of BCL-3

BCL-3 is a proto-oncogene and an atypical inhibitory member of the IκB family [85]. Unlike other members of the IκB family, BCL-3 contains seven ankyrin repeat domains and a C-terminal transactivation domain, enabling both inhibitory and transcriptional regulatory functions [173,174]. Its subcellular localization is context-dependent; however, BCL-3 predominantly localizes to the nucleus, where it interacts with p50 and p52 homodimers to regulate gene transcription, thereby affecting various cellular functions [168]. BCL-3 was initially identified on chromosome 19 at the site of recurring translocation t (14; 19) in patients with chronic lymphocytic leukemia, and its oncogenic role in several B-cell malignancies has been established [175,176]. However, the functional relevance of BCL-3 in PC has not been thoroughly investigated, with limited data available regarding its specificity, regulatory mechanism, and contribution to treatment resistance, highlighting a significant gap in current knowledge.

**Figure 5:**
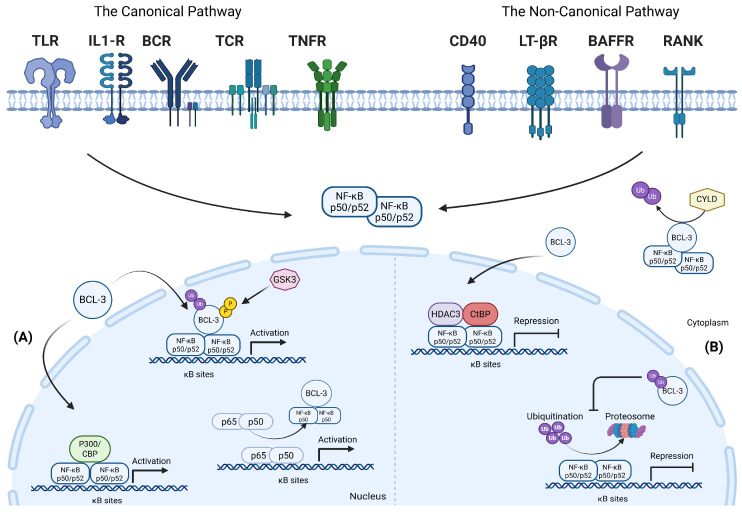
BCL-3 bifunctional role within the NF-κB pathway. BCL-3 acts as either a transcriptional co-activator or co-repressor of NF-κB target genes, with its function regulated by upstream signaling, post-translational modifications, and the cellular environment. (**A**) As a co-activator, BCL-3 interacts with NF-κB p50 or p52 homodimers at κB DNA elements, enhancing their DNA binding stability and providing transactivation domains that are lacking in these homodimers. BCL-3 also recruits transcriptional co-activators such as p300/CBP to enhance NF-κB target gene expression. BCL-3 can also remove inhibitory p50 homodimers from κB sites, allowing NF-κB heterodimers to promote transcription. GSK3-mediated phosphorylation modulates the nuclear location and transcriptional function of BCL-3. (**B**) As a co-repressor, BCL-3 binds p50 or p52 homodimers and recruits co-repressors such as HDACs and CtBP. BCL-3 additionally inhibits the proteasomal degradation of p50/p52, hence prolonging their repressive function. CYLD modulates BCL-3 stability and nuclear localization by cleaving polyubiquitin chains, thus influencing its ability for transcriptional activation or repression. Created in BioRender. Alhakm, R. (2026) https://BioRender.com/4o18riu (accessed on 12 March 2026).

BCL-3 plays a dual regulatory role in the NF-κB pathway through direct interactions with NF-κB subunits, acting as either a transcriptional repressor or activator depending on cellular context, promoter composition, and extracellular stimuli [17,85] (Figure 5). BCL-3 acts as a negative feedback regulator of the NF-κB pathway, maintaining cellular homeostasis by preventing excessive NF-κB activation [177,178]. Upon TLR and TNFR signaling, it interacts with NF-κB subunits to suppress their nuclear translocation and subsequent DNA binding, thereby inhibiting downstream transcription of pro-inflammatory genes, thus limiting an excessive inflammatory response [179,180]. Additionally, BCL-3 stabilizes inhibitory NF-κB complexes by preventing ubiquitin-mediated degradation, further controlling the inflammatory response [178]. Besides interacting with NF-κB subunits, BCL-3 also interacts with various transcriptional regulators, including the recruitment of co-repressors such as HDACs and C-terminal binding protein (CtBP). These interactions further enhance BCL-3’s transcriptional repression of NF-κB target genes such as CXCL2, CXCL1, IL-6, CCL2, and TNF-α, and contribute to its oncogenic functions, including inhibition of apoptosis [181,182].

Furthermore, BCL-3 functions as a nuclear transcription cofactor that promotes NF-κB-dependent gene expression by the removal of NF-κB inhibitory subunits from gene promoters and provides a transactivation domain of key cell cycle regulators such as Cyclin D1, which drives cell proliferation [183,184]. It also provides the transactivation domain needed for the recruitment of transcriptional co-regulators and co-activators, thereby enhancing the transcription of target genes [185]. Through these interactions, BCL-3 integrates inflammatory and metabolic signaling networks, reinforcing its role as a context-dependent transcriptional regulator with oncogenic potential.

#### Post-Translational Regulation of BCL-3

BCL-3 activity is dynamic and tightly regulated by PTMs, notably phosphorylation and ubiquitination, which regulate its stability, intracellular localization, and oncogenic activity [181,186,187]. These PTMs create a complex regulatory network that determines whether BCL-3 acts as a tumor suppressor or an oncogenic driver.

Phosphorylation functions as a critical molecular switch; phosphorylated BCL-3 translocates to the nucleus and acts as a transcriptional coactivator, enhancing NF-κB-dependent gene transcription activity. Conversely, the non-phosphorylated form of BCL-3 remains in the cytoplasm, acting as an IκB-like inhibitor by sequestering p50 and p52 homodimers, thus restricting NF-κB-mediated transcriptional activity [186,188,189]. Notably, BCL-3 predominantly exists in its phosphorylated form in many cancer cells, making nuclear BCL-3 a marker of activated oncogenic signaling [166,181]. Additionally, ubiquitination provides an additional, independent layer of regulation by controlling BCL-3 stability and nuclear translocation, thereby modulating NF-κB-dependent transcription. The deubiquitinase CYLD, a tumor suppressor and negative regulator of various signaling pathways, removes ubiquitin chains from BCL-3, hence preventing its nuclear translocation and accumulation and suppressing the subsequent expression of NF-κB target genes involved in cell proliferation [190]. Mutations in genes involved in the ubiquitination machinery, particularly CYLD, can disrupt protein homeostasis and signaling pathways, thereby contributing to cancer progression [191,192] (Figure 5). Building on the post-translational regulation of BCL-3, its interaction with the NF-κB pathway creates an autoregulatory feedback loop where NF-κB activation upregulates BCL-3 transcription. In turn, phosphorylated BCL-3 enhances NF-κB signaling by promoting the expression of target genes involved in oncogenesis and inflammation. This BCL-3/NF-κB feedback loop adds a layer of complexity to the regulatory dynamics of NF-κB signaling, particularly influencing the type of NF-κB dimers involved in the signaling cascade and the specificity of downstream gene expression [177]. Disruption of this regulatory axis contributes to BCL-3 dysregulation.

### 6.3. BCL-3-Mediated Regulation of NF-κB-Dependent Apoptosis

Apoptotic dysregulation is a hallmark of cancer progression and therapy resistance; however, the contribution of BCL-3 to apoptotic signaling remains poorly characterized in PC. Evidence from other cancer models demonstrates that BCL-3 has emerged as a context-dependent regulator of apoptosis, exerting both pro-apoptotic and anti-apoptotic effects through direct and indirect interactions with key apoptotic regulators, including but not limited to BCL-2. A study in breast cancer cells demonstrated that BCL3 with p50 and p52 homodimers binds to specific κB sites within the BCL2 promoter, thereby enhancing BCL-2 transcription activation and supporting cell survival [193].

Despite these predominantly pro-survival functions, in multiple myeloma (MM) cells, BCL-3 overexpression induces apoptosis, while its knockdown does not significantly affect MM cell viability, underscoring the complexity of its function [194]. Collectively, these findings highlight the high complexity, context-dependent, and multifaceted role of BCL-3 in apoptotic regulation across different cancer types. However, mechanistic studies exploring its role in PC are notably lacking, particularly to determine how BCL-3 regulates NF-κB-dependent anti-apoptotic genes in PC. Among these targets, BCL-2 is a well-established NF-κB downstream target with a key role in apoptosis and therapy resistance, highlighting a potential mechanistic axis through which BCL3 may regulate cell survival.

#### 6.3.1. BCL-2 Function and Apoptosis Regulation Within NF-*κ*B Signaling

BCL-2 is a key anti-apoptotic member of the BCL-2 protein family, localized on the mitochondrial outer membrane, and widely recognized for its role in promoting cell survival via controlling the intrinsic apoptosis pathway. The regulation of BCL-2 activity involves multiple mechanisms that significantly impact its anti-apoptotic function. PTMs play a key role in modulating BCL-2 interactions with other proteins, influencing the conformational state and the formation of functional protein complexes [195,196]. Moreover, the expression of BCL-2 is tightly regulated by the activity of various transcription factors, such as p53 [197], WT1 [198], NF-*κ*B [153], and STATs [199], as well as by epigenetic modifications [200]. Notably, NF-κB activation drives BCL-2 overexpression, linking oncogenic signaling to apoptosis resistance and cancer progression [153,201,202]. Unlike other mechanisms that alter the metabolism or pharmacokinetics of anticancer drugs, BCL-2 regulates apoptosis by blocking the transmission of damage-induced apoptotic signals to downstream effectors. This prolonged cellular survival under cytotoxic stress increases the probability of acquiring genetic mutations, which ultimately contribute to disease progression, metastatic potential, and resistance to therapies [163,203,204].

#### 6.3.2. Role of BCL-2 in Prostate Cancer in the Context of NF-κB

BCL-2 is a key anti-apoptotic regulator that maintains mitochondrial integrity and promotes tumor cell survival in multiple cancers, including PC [205,206,207]. In normal prostate tissue, BCL-2 expression is restricted to basal epithelial cells, while its upregulation in PIN suggests early involvement in tumorigenesis [208,209]. In PC, BCL-2 overexpression enhances survival of both primary and metastatic cells and contributes to resistance to androgen deprivation therapy (ADT) and other anticancer treatments, including chemotherapy and radiotherapy [153,208,210]. Modulation of BCL-2 protein expression has been shown to improve the efficacy of these therapies [210,211,212,213]. Clinically, elevated BCL-2 levels have been associated with adverse pathological features [214,215,216]. Conflictingly, an inconsistent correlation was reported by another study, as no significant correlation between BCL-2 overexpression and either Gleason score or tumor stage was observed [217]. These interstudy discrepancies may reflect tumor heterogeneity and variations in patient demographics, highlighting the complexity of PC biology and suggesting that BCL-2 alone may be insufficient as a reliable prognostic marker. However, its biological relevance may be better understood and accurately interpreted by investigating its interactions with additional molecular markers, including NF-κB regulatory proteins.

Several signaling pathways are involved in the regulation of BCL-2 activity and PC progression. Evidence suggests that BCL-2 acts as a downstream target of NF-κB in PC, where TNFα enhances NF-κB/p65 and p50 binding to the BCL-2 P2 promoter in LNCaP cells, driving BCL-2 overexpression [153]. Furthermore, another study confirms that NF-κB activation can inhibit TNFα-induced pro-apoptotic signaling in PC cells [218]. Notably, the relation between TNFα-mediated BCL-2 overexpression and NF-κB activity is hormone-dependent, reflecting context-specific control of BCL-2 in PC. Given that BCL-3 also has a role in NF-κB signaling and apoptosis regulation, it may modulate BCL-2 expression, highlighting a potential crosstalk between these two key regulators in PC.

## 7. Emerging Regulatory Axis: Potential Interplay Between BCL-3 and BCL-2

BCL-2 and BCL-3 are key regulators in PC progression, acting through distinct yet potentially interconnected mechanisms. BCL-2 inhibits apoptosis as a downstream target of NF-κB, contributing to therapy resistance and tumor cell survival. BCL-3, a nuclear IκB protein, modulates NF-κB transcriptional activity by interacting with p50 and p52 homodimers in various cancers in a context-dependent manner. However, the regulatory relationship between BCL-3 and BCL-2 in PC remains unexplored with no direct evidence supporting a role for BCL-3 in regulating BCL-2 expression in PC. Furthermore, it remains unknown whether BCL-3 directly binds to the BCL-2 promoter, regulates its expression, or impacts apoptosis and therapy resistance in PC cells through NF-κB-dependent mechanisms.

Evidence from other biological systems provides indirect support for a potential functional link between these molecules. A breast cancer model demonstrates that BCL-3, in association with p50 and p52 homodimers, acts as a critical regulator of anti-apoptotic signaling, suggesting a potential mechanism by which BCL-3 might contribute to therapy resistance [193]. While this mechanism has not been investigated in PC, it could suggest a potential mechanism by which BCL-3 could similarly modulate BCL-2 expression in PC, thereby influencing apoptotic pathways and contributing to therapy resistance.

Based on these observations, we propose a hypothesis-driven model in which BCL-3 may act as a context-dependent regulator of NF-κB-mediated survival signaling, with the potential to influence BCL-2 expression. In particular, we hypothesize that a BCL-3/BCL-2 regulatory axis may influence disease progression in PC. To investigate this, several experimental approaches could be considered, including modulation of BCL-3 expression via knockout or overexpression in various PC cell lines, followed by assessment of BCL-2 expression and other apoptotic markers. Chromatin immunoprecipitation (ChIP) assays could further determine whether BCL-3 directly binds to the BCL-2 promoter in PC cells. To further assess the functional impact of this interaction, treatment with chemotherapeutic agents or ADT, followed by apoptosis assays, needs to be done to determine the impact of BCL-3 modulation on cell survival. In vivo studies using xenograft models with BCL-3-deficient cells or mice could provide additional insights into potential contribution of this axis in tumor growth, apoptosis resistance, and therapeutic response. Investigating the potential BCL-3/BCL-2 regulatory axis in PC could reveal novel mechanisms of apoptosis regulation and identify promising therapeutic targets; however, its functional relevance remains to be established.

## 8. Therapeutic Implications and Translational Perspectives

Despite extensive research and advances in therapeutic approaches, management of PC remains challenging, as available treatments are not fully curative and primarily aim to prolong patient survival. PC treatment. Disease management remains challenging due to tumor heterogeneity, lack of molecular target therapy, resistance to treatment, and post-therapeutic complications [5,16,219]. There is significant clinical heterogeneity among PC patients, with unique molecular characteristics in each case. Therefore, a crucial issue in the therapeutic management of PC is the identification and prediction of biomarkers to develop effective treatments against the different groups of PC patients.

### 8.1. Clinical Management of Prostate Cancer

**Figure 6:**
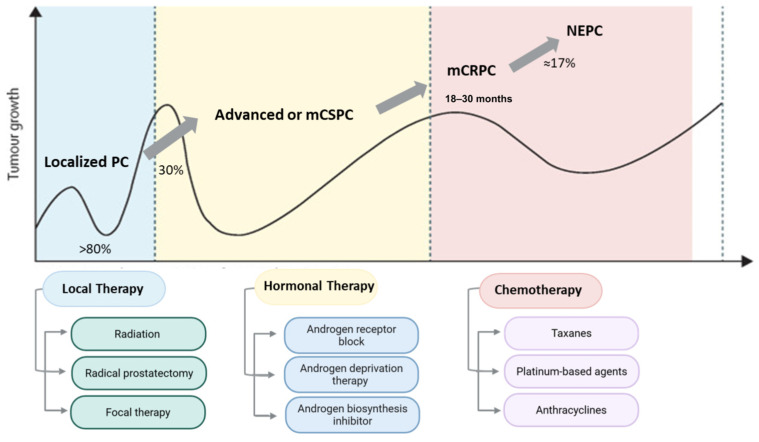
Therapeutic strategies for prostate cancer management. Schematic representation of the main therapeutic approaches in prostate cancer, including active local therapies, hormonal therapy, and systemic chemotherapy used in PC. Created in BioRender. Alhakm, R. (2026) https://BioRender.com/pis6cgz (accessed on 12 March 2026).

The management of PC is guided by a combination of clinical and pathological factors shaping the treatment plan. Key factors include PSA levels, tumor stage, histological grade, and Gleason score. In the early stages of the disease, elderly patients or those with health comorbidities are often managed with an active surveillance approach, which involves close monitoring of PSA levels and a periodic Digital Rectal Examination (DRE) to assess disease progression. For patients with localized disease, active local therapy, such as radiation and radical prostatectomy, remains a standard treatment option for over 80% of the cases, despite the potential side effects associated with these treatments [5,6]. Additionally, focal therapy with TOOKAD^®^ (padeliporfin-based vascular-targeted photodynamic therapy) has shown promise in delaying progression to high-grade disease compared to standard surveillance [220,221]. Nonetheless, approximately 20–40% of cases eventually progress to a metastatic state, forming castration-sensitive prostate cancer cases (CSPC), which are mainly managed with hormonal therapy (Figure 6).

Hormonal therapies are widely used to suppress androgen signaling, a key driver of PC progression. Those include ADT, which targets androgen production from the testicles; examples include surgical castration, luteinizing hormone-releasing hormone (LHRH) agonists (e.g., leuprolide), LHRH antagonists (e.g., degarelix), and gonadotropin-releasing hormone (GnRH) agonists or antagonists [222,223]. Additional hormonal therapies inhibit androgen synthesis from other parts of the body, including the adrenal glands and PC cells, such as the androgen biosynthesis inhibitor Abiraterone, which suppresses CYP17A1 enzymatic activity. Moreover, AR signaling can be directly blocked by AR antagonists, such as Flutamide, a first-generation AR antagonist, and Enzalutamide, a next-generation AR antagonist that alters AR nuclear translocation and binding to androgen response elements on DNA (Figure 6).

These drugs are frequently administered in combination with chemotherapy to enhance therapeutic efficacy, particularly in advanced disease. Taxane-based chemotherapeutic agents, such as docetaxel or cabazitaxel, are commonly used and exert their anticancer effects by disrupting microtubule dynamics, thereby inhibiting mitosis and suppressing PC cell proliferation. Furthermore, taxane-based chemotherapeutic agents such as docetaxel have been administered in combination with thymoquinone, a potent antioxidant phytochemical. This combinational treatment induces apoptosis in PC through PI3K/Akt suppression. IL-7 increases the PC progression, and administration of thymoquinone results in the suppression of Akt/NF-kB axis, thus preventing IL-7 oncogenic activity and reducing PC metastasis by reducing MMP-3 and MMP-7 levels [224].

Additional chemotherapeutic agents, including platinum-based compounds (e.g., cisplatin) and anthracyclines (e.g., doxorubicin), have been explored in advanced cases. Platinum-based compounds such as cisplatin are activated within the cell, where they form DNA cross-links, resulting in structural damage, disrupting DNA replication and transcription; this structural damage results in the activation of p53, leading to cell cycle arrest [225]. Furthermore, anthracyclines such as doxorubicin exhibit cytotoxic effects by inhibiting cell proliferation and survival. This is achieved through various mechanisms, including DNA intercalation, reactive oxygen species production, and topoisomerase II inhibition, ultimately leading to double-strand DNA breaks [226]. However, despite the initial response, most patients eventually develop resistance within approximately 18–30 months, leading to the emergence of CRPC and, in a subset of cases, toward a neuroendocrine prostate cancer (NEPC), which is characterized by poor prognosis and limited therapeutic options (Figure 6) [5,6].

### 8.2. Limitations of Direct NF-κB Targeting

The aggressive progression of PC and the emergence of treatment resistance highlight the need to explore alternative therapeutic strategies targeting key signaling pathways that drive PC progression, including the NF-κB pathway. However, therapeutic targeting of NF-κB remains challenging due to its ubiquitous activity and essential roles in normal cellular homeostasis, immune responses, and broader physiological processes. Thus, systemic inhibition of the pathway may disrupt essential cellular processes and lead to significant toxicity, limiting its clinical applicability [227]. In addition, compensatory signaling mechanisms further reduce the effectiveness of single-agent NF-κB inhibition strategies. Consequently, most therapeutic strategies have focused on indirect modulation of pathway, including targeting upstream regulators such as IKK complex, inhibiting proteasome-mediated degradation of IκB, or modulation of signaling pathways that converge on NF-κB activation.

Several drugs have been investigated for their potential to target the NF-κB pathway in the context of PC, including proteasome inhibitors, IKK inhibitors, natural compounds and anti-inflammatory drugs. However, the complexity of this pathway has limited their clinical effectiveness. For example, the proteasome inhibitor, Bortezomib, prevents IκB degradation and thereby inhibits NF-κB activation; however, its clinical efficacy in PC has been limited, partly due to compensatory stabilization of NIK which leads to the reactivation of the NF-κB pathway [228,229]. Resistance to bortezomib can arise from variations in proteasome subunits, alterations in key components of the NF-κB pathway, or the development of alternative survival pathways [228,229]. Similarly, pharmacological IKK inhibitors such as BMS-345541 and CmpdA suppress proliferation and migration, highlighting their therapeutic potential in targeting advanced PC, with evidence limited to preclinical studies [138,230]. Additionally, parthenolide has been shown in preclinical models to target IKK, thereby inhibiting NF-κB signaling in PC cells. This results in reduced cell viability and enhanced sensitivity to chemotherapeutic and radiosensitizing agents [231,232]. However, these findings are limited to in vitro and animal studies, and parthenolide has not yet progressed to clinical studies in PC.

Furthermore, andrographolide and thalidomide exhibit anti-inflammatory properties that can modulate the NF-κB pathway in the context of PC [233,234]. While andrographolide has shown promising anti-tumor and NFκB inhibitory effects in preclinical PC models, it has not yet been evaluated in clinical trials. In contrast, thalidomide has been evaluated in a completed phase II clinical study in PC patients in which 27% of patients experienced a ≥40% decline in PSA, supporting further investigations of thalidomide as a potential therapeutic target in combination with other therapies (Table 1) [235].

Natural compounds such as curcumin and apigenin have shown potential in targeting NF-κB signaling in PC. Curcumin, derived from turmeric, can inhibit NF-κB signaling and reduce pro-inflammatory effects in PC. It is currently being evaluated in a Phase III clinical trial (NCT03769766) in men with low-risk prostate cancer undergoing active surveillance. However, its rapid metabolism and low bioavailability may limit its therapeutic efficacy [236,237,238]. Similarly, apigenin has been shown in preclinical studies to repress the expression of NF-κB-regulated genes, including BCL-2, cyclin D1, COX-2, and VEGF, while enhancing the sensitivity of PC cells to TNF-α-induced apoptosis [239]. Despite these findings, apigenin has not yet been evaluated in clinical studies. Further NF-κB-targeted therapies for PC patients include a combination of aspirin, a non-steroid anti-inflammatory drug, and levofloxacin, an antibiotic that inhibits TLR-4 signaling. This therapeutic approach is still in clinical trial, where it requires evaluation in larger patient cohorts with extended follow-up sessions to achieve firm findings [240,241].

While NF-κB-targeting compounds have shown promise, the development of resistance to these drugs remains a major limitation, arising from altered drug metabolism, limited bioavailability, genetic or functional alterations in NF-κB pathway components, or upregulation of alternative survival pathways. These challenges reflect the highly dynamic and context-dependent nature of NF-κB signaling, which operates through multiple regulatory nodes rather than a single linear pathway. Consequently, global pathway inhibition is unlikely to improve therapeutic efficacy and may disrupt physiological processes. This highlights the need for more selective approaches that target NF-κB signaling at the level of transcriptional specificity or downstream effectors. In this context, targeting NF-κB-dependent transcriptional outputs associated with tumor progression and survival may provide a more effective strategy. In PC, anti-apoptotic signaling represents a key downstream consequence of NF-κB activation. Therefore, co-targeting survival pathways such as BCL-2, rather than the pathway as a whole, may provide a more precise therapeutic approach. Consistent with this, combined inhibition of IKKβ and BCL-2 has been shown to delay the development of resistance to Enzalutamide in preclinical models [242].

Therefore, temporal and dynamic targeting of NF-κB may allow modulation of this pathway at specific stages of prostate cancer progression while limiting compensatory resistance mechanisms. In this context, the previously discussed interplay between BCL-2 and BCL-3 represents a relevant downstream therapeutic axis. While multiple BCL-2 inhibitors have been explored in prostate cancer, BCL-3 remains largely unexplored as a therapeutic target, highlighting a promising direction for future drug development.

**Table 1:** Potential therapeutic strategies.

**Therapeutic Agent**	**Study Model**		**Mechanism of Action**			**Refs.**
Bortezomib	In vitro: PC3, LNCaP					[228]
			It inhibits 26s proteasome activity Inhibits IkB degradation			
BMS-345541	In vitro: PC3					[230]
			Inhibits IkBa phosphorylation and NF-κB/p65			
CmpdA	In vivo: Xenograft with PC3 cells					[138]
			Inhibits the expression of Nanog, Oct-4, and Sox-2			
Parthenolide	In vitro: LNCaP DU 145 PC3					[231]
			Inhibits the NF-κB pathway by targeting the IkB kinase complex			
Androgapholide	In vitro: LNCaP, DU145, PC3		Inhibits IL-6 expression Suppressing IL-6 induced signaling of Akt, Stat3, and ERK1/2			[233]
	In vivo: Human prostate cancer xenografts					
Apigenin	In vitro: PC3					[239]
			Represses the expression of NF-κB-regulated genes, including BCL-2, cyclin D1, COX-2, and VEGF			
**Therapeutic Drugs in Clinical Trials**						
**Therapeutic Agent**	**Study Model**	**Mechanism of Action**		**Clinical Phase**	**Clinical Trials ID**	**Ref** **s** **.**
Curcumin	In vitro: PC3 LNCaP	Inhibits NF-κB signaling Suppresses AR gene transcription Exhibits anti-inflammatory effects		**NA**	**NA**	[236,238]
	In vivo: Clinical studies			Ongoing clinical trials phase 3	NCT03769766	[237]
AT-101	In vivo VCaP prostate cancer xenografts	Blocks BCL-2 through the BH3 domain binding		**NA**	**NA**	[243]
	In vivo: Clinical studies			Phase II clinical trials	NCT00666666	[244]
Oblimersen Sodium + Docetaxel	In vivo: Clinical studies	Substitutes sulfur for non-bridging oxygen molecules in the phosphate backbone, ultimately resulting in BCL-2 inhibition		Phase I clinical trials Phase II clinical trials	NCT00085228.	[245]
Enzalutamide + Venetocla	In vivo: Clinical studies	Promotes BCL-2B generation, simultaneously degrading BCL-2 protein expression		Clinical trials phase Ib	NCT03751436	[246]
Thalidomide	In vivo: Clinical studies	Suppresses inflammatory signaling through downregulation of TNF-α and inhibition of NF-κB pathway activity		Phase II clinical trials	NCT00001446	[234,235]

### 8.3. Downstream and Regulatory Targets

#### 8.3.1. Management of PC via BCL-2

As prostate cancer morbidity and mortality rates continue to rise, researchers are exploring alternative therapeutic approaches to overcome the growing resistance to conventional therapies. BCL-2 has been directly associated with resistance to chemotherapeutic agents in CRPC patients. As mentioned earlier, it has grown as an interesting molecular target, leading to the development and testing of multiple anti-BCL-2 agents.

A small molecule known as AT-101 (R-(-)-gossypol acetic acid), derived from racemic gossypol, was studied for its anti-BCL-2 properties. AT-101 binds to the BH3 domain in BCL-2, blocking apoptosis inhibitors while simultaneously stimulating pro-apoptotic proteins. Before clinical trials, AT-101 was tested in vitro on the VCap human prostate cancer cell line; researchers found that BCL-2 is significantly overexpressed in androgen-independent cells compared to androgen-dependent cells, indicating that depletion of BCL-2 activity can delay resistance. Furthermore, in VCaP prostate cancer xenografts, researchers found that AT-101 is synergistic with androgen deprivation, ultimately leading to decreased tumor volume. Consequently, the researchers decided to carry out clinical trials, where a phase II trial was conducted in men with castration-sensitive metastatic PC treated with AT-101 [244].

Furthermore, another study looked at oblimersen, an 18-base synthetic oligodeoxyribonucleotide strand that hybridizes the first six codons of BCL-2 mRNA, inhibiting the expression of the BCL-2 protein. This antisense agent is resistant to cleavage by extracellular and intracellular nucleases, exhibiting greater stability compared to naive oligonucleotides. Oblimersen substitutes sulfur with non-oxygen-binding molecules in the phosphate backbone; its ability to inhibit BCL-2 leads to enhanced anti-tumor activity. The impact of oblimersen sodium combined with docetaxel on BCL-2 protein expression in CRPC patients has been examined. Researchers found that this regimen combination led to a decrease in BCL-2 expression in peripheral blood mononuclear cells post-treatment. Researchers concluded that Oblimersen combined with docetaxel is an active therapeutic combination for CRPC patients, indicating encouraging response rates and a higher overall median survival, along with minimal toxicities and clear BCL-2 protein inhibition [245].

Another BCL-2 inhibitor studied is venetoclax, which, upon combination with enzalutamide, exhibited strong CRPC-inhibitory effects. Ventoclax has been shown to promote rapid BCL-2B generation, ultimately leading to a simultaneous BCL-2 degradation. Upon the administration of a standard dose of enzalutamide for 4 weeks, venetoclax was administered at doses ranging from 400 to 600 mg. The phase Ib combination trial concluded that enzalutamide and venetoclax treatment have an acceptable toxicity profile, and a clinical response was elicited in patient subsets [246]. Although BCL-2 targeted therapies have shown promise in PC, the emergence of treatment resistance highlights the need to explore additional regulators of tumor cell survival mechanisms. Targeting regulatory molecules that influence BCL-2 expression may therefore enhance therapeutic efficacy. However, therapeutic targeting of BCL-2 is associated with important limitations. Given its essential role in normal hematopoietic and immune cell survival, BCL-2 inhibition may result in on-target toxicities, including disruption of immune homeostasis. In addition, redundancy within pro-survival signaling networks may attenuate therapeutic efficacy, as compensatory upregulation of alternative anti-apoptotic proteins such as MCL-1 or BCL-XL can sustain tumor cell survival. BCL-3 has been shown to affect survival signaling and tumor progression in various cancers and to regulate BCL-2 expression; consequently, further investigation into the therapeutic potential of targeting BCL-3 in PC is crucial.

#### 8.3.2. Therapeutic Potential of Targeting BCL-3 in Prostate Cancer

BCL-3, as discussed earlier, plays critical roles in multiple oncogenic signaling pathways and can influence therapy response and contribute to drug resistance, making it a potential therapeutic target. Nevertheless, targeting BCL-3 presents additional challenges due to its role as a transcriptional co-regulator of NF-κB. Modulation of BCL-3 may lead to broad alterations in NF-κB-dependent transcriptional programs, potentially affecting inflammatory signaling and cellular stress responses. Furthermore, the context-dependent nature of NF-κB signaling may result in variable or even opposing biological effects across different tumor settings. BCL-3 suppression could effectively inhibit cancer progression, and studies in BCL-3 knockout mice show that loss of BCL-3 exhibits only a mild immune defect compared to knockouts of other NF-κB subunits, suggesting that targeting BCL-3 may achieve anti-tumor effects without significant toxicity or with a minimal side effect [247,248].

Direct evidence supporting a role for BCL-3 in PC therapy resistance emerged in 2013. In this study, elevated BCL-3 expression in PC cells was associated with reduced sensitivity to chemotherapeutic agents, including staurosporine, etoposide, and paclitaxel. Suppression of BCL-3, using shRNA in DU145 xenograft models, significantly reduced tumor growth and enhanced apoptosis, as indicated by increased cleaved caspase-3, without affecting the mitotic index. Mechanistically, BCL-3 binds NF-κB sites in the promoters of the Helix-Loop-Helix proteins Id1 and Id2, mediating anti-apoptotic effects. Moreover, IL-6 stimulation upregulated BCL-3, protecting PC cells from apoptosis and further supporting its role in therapy resistance [172]. A promising drug targeting the BCL-3/NF-κB signaling pathway is currently being investigated for its effectiveness in PC, with a focus on aggressive forms of the disease; no further information was available online [249].

Although studies in PC are still limited, evidence from other cancers supports the broader therapeutic relevance of BCL-3. Researchers have demonstrated the therapeutic potential of targeting BCL-3 in cancer [96]. In breast cancer models, a small-molecule inhibitor disrupting BCL-3, p50 binding, effectively reduced tumor growth and metastasis [250]. Similarly, in metastatic melanoma, BCL-3 inhibition effectively suppressed cyclin D1 expression and cancer cell proliferation [251]. These studies further highlight its potential as a viable therapeutic strategy. Additional potential strategies may involve targeting specific modifications of BCL-3, such as post-translational modifications or the upstream regulators, to modulate its activity [96]. While BCL-3 is a key regulator of the NF-κB pathway, complete depletion of its activity does not fully eliminate the NF-κB signaling. Moreover, the precise mechanism by which BCL-3 regulates the NF-κB pathway under different conditions is not yet fully understood. In addition, it is not yet clear whether BCL-3 functions as a stable molecule during disease progression or undergoes dynamic translocation at different stages of the disease.

Although emerging evidence emphasizes the significance of BCL-3 in cancer, including PC, a significant gap in knowledge remains in understanding its exact role in the progression of PC, therapy resistance, and its potential as a combination therapeutic target aimed to improve efficacy and overcome resistance. Specifically, the mechanism by which BCL-3 regulates NF-κB signaling and promotes oncogenic processes in the context of PC remains poorly characterized. From a therapeutic perspective, targeting BCL-3 may attenuate oncogenic NF-κB activity while also disrupting tumor survival pathways mediated by anti-apoptotic proteins such as BCL-2; however, this hypothesis requires further validation. Furthermore, it remains unclear whether the oncogenic effects of BCL-3 are maintained throughout the progression of PC or dynamically regulated in a stage-specific way. Addressing these concerns may shed light on the identification of new therapeutic targets for PC.

## 9. Conclusions and Future Perspectives

PC progression is increasingly understood as a dynamic process driven by chronic inflammation and sustained immune dysregulation within the tumor microenvironment. Among the inflammatory signaling networks implicated in this process, the NF-κB pathway plays a central role by integrating extracellular inflammatory cues with transcriptional programs that promote cell survival, proliferation, and resistance to therapy. Although NF-κB dysregulation has been widely reported in PC, its regulatory complexity and context-dependent functions across disease stages remain incompletely resolved, limiting precise mechanistic characterization and the identification of stage-specific therapeutic targets.

This review synthesizes current evidence supporting constitutive NF-κB activation in PC, particularly in advanced and castration-resistant disease, and highlights how dysregulation across multiple regulatory layers contributes to persistent oncogenic signaling. While the anti-apoptotic protein BCL-2 is well established as a downstream effector of NF-κB and a mediator of apoptosis resistance in PC, the functional role of BCL-3 in shaping NF-κB transcriptional output in this disease remains poorly defined. Existing data from other malignancies suggest that BCL-3 can act as a context-dependent modulator of NF-κB activity and influence the expression of pro-survival genes, including BCL-2; however, direct mechanistic validation of this regulatory axis in PC is currently lacking. Thus, we propose a testable, hypothesis-driven framework in which BCL-3 may act as a context-dependent regulatory node that may fine-tune NF-κB-dependent survival signaling in PC. In this context, a potential BCL-3/BCL-2 regulatory axis may contribute to disease progression, although this remains speculative and requires experimental validation. Determining whether BCL-3 directly modulates BCL-2 expression or broader apoptotic programs in PC will require focused experimental approaches, including chromatin-based analyses, functional perturbation studies in clinically relevant cell models, and in vivo validation.

From a translational perspective, NF-κB’s complex regulatory architecture may explain the limited clinical success of direct pathway inhibition strategies. Targeting downstream regulatory nodes that shape NF-κB transcriptional specificity, such as BCL-3 or BCL-2, may offer a more selective and stage-adapted therapeutic approach, particularly in advanced and castration-resistant prostate cancer. Future studies integrating molecular, pathological, and clinical data will be essential to determine whether modulation of NF-κB regulatory dynamics can be leveraged to overcome therapy resistance and improve patient outcomes.

## Data Availability

No new data were created or analyzed in this study. Data sharing is not applicable.

## Abbreviations

The following abbreviations are used in this manuscript:

ADAM	A Disintegrin and Metalloprotease
ADAMTS1	A Disintegrin and Metalloproteinase with Thrombospondin Type 1 Motif 1
ADT	Androgen deprivation therapy
AIPC	Androgen-Independent Prostate Cancer
AKT	Serine/threonine kinase (Protein Kinase B)
AMACR	α-methyl-acyl-CoA racemase
AR	Androgen receptor
Bak	Bcl-2 homologous antagonist/killer
BAX	BCL2-Associated X, Apoptosis Regulator
BCL-10	B-Cell Lymphoma 10
BCL-2	B-Cell Lymphoma-2
BCL-3	B-Cell Lymphoma-3
BCL-XL/BCL2L1	B-Cell Lymphoma-Like 1
BCR/TCR	B-cell/T-cell receptors
BPH	Benign Prostatic Hyperplasia
CARD11	Caspase Recruitment Domain-Containing Protein 11
CBM Complex	CARD11–BCL10–MALT1 complex
CBP/p300	CREB-Binding Protein/E1A-Binding Protein p300
CCL19	C-C motif chemokine ligand 19
CCL21	C-C motif chemokine ligand 21
CCND1	Cyclin D1
CCND2	Cyclin D2
c-Fos	Fos proto-oncogene, AP-1 transcription factor subunit
c-Jun	Jun proto-oncogene, AP-1 transcription factor subunit
CmpdA	Compound A
COX-2	Cyclooxygenase-2
c-Rel	REL Proto-Oncogene, NF-KB Subunit
CRPC	Castration-resistant prostate cancer
CtBP	C-terminal binding protein
CWR22rv1	Castration-resistant prostate cancer cell line derived from CWR22 xenograft
CXCL13	C-X-C motif chemokine ligand 13
CXCR4	C-X-C motif chemokine receptor 4
CYLD	Cylindromatosis (lysine 63 deubiquitinase)
CYP17A1	Cytochrome P450 Family 17 Subfamily A Member 1
DRE	Digital Rectal Examination
EGFR	Epidermal Growth Factor Receptor
EMT	Epithelial-to-mesenchymal transition
ErbB2	Erb-B2 Receptor Tyrosine Kinase 2
ERG	ETS-related gene
ERK 1/2	Extracellular signal-regulated kinases 1 and 2
FOXOs	Forkhead box O transcription factors
GEO	Gene Expression Omnibus
GnRH	Gonadotropin-releasing hormone
GSK3β	Glycogen Synthase Kinase-3 beta
GSTP1	Glutathione S Transferase Pi1
GTEx	Genotype-Tissue Expression
HDACs	Histone Deacetylases
HDM2/MDM2	MDM2 proto-oncogene
HIF-1α	Hypoxia-Inducible Factor-1 alpha
HPV	Human papillomavirus
IAP1	Inhibitor of Apoptosis 1
IAP2	Inhibitor of Apoptosis 2
ICAM-2/5	Intercellular Adhesion Molecule 2/5
IDO1	Indoleamine 2,3-Dioxygenase 1
IFN-γ	Interferon-Gamma
IKK complex	IκB kinase (κB kinase) complex
IKKα	IκB kinase alpha
IKKβ	IκB kinase beta
IKKγ/NEMO	IκB kinase gamma/NF-κB Essential Modulator
IL-1R	Interleukin-1 receptor
IL-1α	Interleukin-1 alpha
IL-1β	Interleukin-1 beta
IL6	Interleukin-6
IL8	Interleukin-8
IRF4	Interferon Regulatory Factor 4
IκB	Inhibitor κB
IκBNS	Inhibitor of κB, nuclear site
IκBα	Inhibitor of κB alpha
IκBβ	Inhibitor of κB beta
IκBε	Inhibitor of κB epsilon
IκBζ	Inhibitor of κB zeta
JAK/STAT3	Janus Kinase/Signal Transducer and Activator of Transcription 3
Ki-67	Marker of Proliferation Ki-67
LHRH	Luteinizing hormone-releasing hormone
LNCaP	Lymph Node Carcinoma of the Prostate
LT	Lymphotoxin
LTβR	Lymphotoxin Beta Receptor
MALT1	Mucosa-Associated Lymphoid Tissue Lymphoma Translocation Protein 1
MAPK	Mitogen-Activated Protein Kinase
MCP-1	Monocyte Chemoattractant Protein-1
MDA-PCa-2b	Prostate carcinoma cell line (bone metastasis-derived)
MDSCs	Myeloid-derived suppressor cells
MM	Multiple myeloma
MMP3	Matrix metallopeptidase 3
MMP7	Matrix metallopeptidase 7
MMP9	Matrix metallopeptidase 9
MYC	MYC proto-oncogene
MyD88	Myeloid Differentiation Primary Response 88
NANOG	Homeobox Transcription Factor Nanog
NF-κB	Nuclear factor kappa B
NF-Κb/Rel	NF-κB/Rel family of transcription factors
NF-κB1 (p105)	Nuclear factor kappa B subunit 1
NF-κB2 (p100)	Nuclear factor kappa B subunit 2
NIK	NF-κB-inducing kinase
NO	Nitric Oxide
Oct4	Octamer-binding transcription factor 4
OX40/TNFRSF4	OX40 receptor/TNF Receptor Superfamily Member 4
p100	NF-κB2 precursor protein
p105	NF-κB1 precursor protein
p50	Processed subunit of NF-κB1 (derived from p105)
p52	Processed subunit of NF-κB2 (derived from p100)
PC	Prostate cancer
PC3	Human Prostate Cancer Cell Line derived from a metastatic adenocarcinoma
PD-L1	Programmed Death-Ligand 1
PGE2	Prostaglandin E2
PI3K-AKT-mTOR	Phosphoinositide 3-Kinase—AKT (Protein Kinase B)—Mechanistic Target of Rapamycin
PIN	Prostatic intraepithelial neoplasia
PKCε	Protein Kinase C epsilon
POU4F1	POU Class 4 Homeobox 1
PPARγ	Peroxisome Proliferator-Activated Receptor Gamma
PRRs	Pattern recognition receptors
PSA	Prostate-Specific Antigen
PSMA	Prostate-Specific Membrane Antigen
PTEN	Phosphatase and Tensin Homolog
PTMs	Post-translational modifications
RelA (p65)	v-rel avian reticuloendotheliosis viral oncogene homolog A
RelB	v-rel avian reticuloendotheliosis viral oncogene homolog B
RHBDD1	Rhomboid Domain Containing 1
RHD	Rel Homology Domain
ROS	Reactive oxygen species
SLC43A1	Solute carrier family 43-member 1
Smac	Second Mitochondria-derived Activator of Caspases
SOX2	SRY-Box Transcription Factor 2
STAT3	Signal Transducer and Activator of Transcription 3
TAMs	Tumor-associated macrophages
TCGA	The Cancer Genome Atlas
TGF-β1	Transforming Growth Factor-Beta 1
Th1	T helper type 1 cells
Th17	T helper type 17 cells
TLR3	Toll-Like Receptor 3
TLR4	Toll-Like Receptor 4
TLR9	Toll-Like Receptor 9
TLRs	Toll-Like Receptors
TME	Tumor microenvironment
TMPRSS2-ERG	Transmembrane serine protease 2-ETS-related gene
TNF	Tumor necrosis factor
TNFAIP3	Tumor necrosis factor alpha-induced protein 3
TNFR	Tumor necrosis factor receptor
TNF-α/NF-κB	Tumor necrosis factor alpha/nuclear factor kappa-light-chain-enhancer of activated B cells
TOOKAD	Padeliporfin-based vascular-targeted photodynamic therapy
TRADD	TNF Receptor-Associated Death Domain
TRAFs	TNF Receptor-Associated Factors
Tregs	Regulatory T cells
TRIFs	TIR-domain-containing adaptor-inducing interferon-β
VCAM-1	Vascular Cell Adhesion Molecule 1
VEGF	Vascular Endothelial Growth Factor
WT1	Wilms Tumor 1
